# Molecular diversity of yam virus Y and identification of banana mild mosaic virus isolates infecting yam (*Dioscorea* spp.)

**DOI:** 10.1007/s00705-023-05809-3

**Published:** 2023-06-13

**Authors:** Mame Boucar Diouf, Sébastien Guyader, Marie-Michelle Nopoly, Olyvia Gaspard, Denis Filloux, Thierry Candresse, Armelle Marais, Pierre-Yves Teycheney, Marie Umber

**Affiliations:** 1 INRAE, UR ASTRO, 97170 Petit‑Bourg, Guadeloupe France; 2 CIRAD, UMR AGAP Institut, 97130 Capesterre Belle Eau, France; 3 UMR AGAP Institut, Univ Montpellier, CIRAD, INRAE, Institut Agro, 97130 Capesterre Belle Eau, France; 4 CIRAD, UMR PHIM, 34090 Montpellier, France; 5 PHIM Plant Health Institute, Univ Montpellier, CIRAD, INRAE, Institut Agro, IRD, 34090 Montpellier, France; 6 INRAE, Univ. Bordeaux, UMR BFP, CS20032, 33882 Villenave d’Ornon Cedex, France; 7 CIRAD, UMR PVBMT, 97410 Saint-Pierre, La Réunion France; 8 UMR PVBMT, Université de la Réunion, 97410 Saint-Pierre, La Réunion France

## Abstract

Two members of the family *Betaflexiviridae* associated with yam (*Dioscorea* spp.) have been described so far: yam latent virus (YLV) and yam virus Y (YVY). However, their geographical distribution and molecular diversity remain poorly documented. Using a nested RT-PCR assay, we detected YVY in *D. alata, D. bulbifera, D. cayenensis, D. rotundata*, and *D. trifida* in Guadeloupe, and in *D. rotundata* in Côte d'Ivoire, thus extending the known host range of this virus and geographical distribution. Using amplicon sequencing, we determined that the molecular diversity of YVY in the yam samples analyzed in this work ranged between 0.0 and 29.1% and that this diversity is partially geographically structured. We also identified three isolates of banana mild mosaic virus (BanMMV) infecting *D. alata* in Guadeloupe, providing the first evidence for BanMMV infection in yam.

**Supplementary Information:**

The online version contains supplementary material available at 10.1007/s00705-023-05809-3.

Yam (*Dioscorea* spp.) is widely cultivated in the intertropical belt for its nutritional and pharmaceutical properties [1–3] and economic value [4]. Twenty-five viruses infecting yam are currently recognized by the International Committee on Taxonomy of Viruses (ICTV) [5], including one member of family *Betaflexiviridae*, yam latent virus (YLV) [6], which is assigned to genus *Carlavirus*. An additional virus, yam virus Y (YVY) [7], has all the hallmarks of viruses of a member of the family *Betaflexiviridae*, but has not been formally recognized as a member of this family. YLV has been reported only in *Dioscorea opposita* in China, whereas YVY has been reported in *D. rotundata* in germplasm collections in Nigeria and Ghana, where it was found mostly in mixed infections with yam mosaic virus (YMV), a potyvirus [7]. The symptomatology associated with YLV or YVY infection has not been elucidated. Overall, the molecular diversity, host range, geographical distribution, impact on production, and transmission mode of YLV and YVY remain largely unknown.

In order to fill these knowledge gaps, we undertook a comprehensive prevalence survey of yam-infecting *Betaflexiviridae* members using three sampling sources (Supplementary Table S1). The first source, referred to as ‘Guadeloupe’, included 896 leaf samples of the species *D. alata*, *D. bulbifera*, *D. cayenensis*, *D. esculenta*, *D. rotundata*, and *D. trifida* collected between November 2019 and December 2020 from 18 yam fields scattered across Guadeloupe, as described by Diouf *et al.* [8]. Considering that the relationship between leaf symptoms and viral infection in yam is poorly documented, symptoms, or lack thereof, were not taken into consideration during sample collection. A second source, referred to as ‘BRC-TP’, included 406 leaf samples from the same species, collected between 2014 and 2019 from the *in vitro* yam collection maintained by the Biological Resources Center for Tropical Plants (BRC-TP) in Guadeloupe [9]. A third and last source, referred to as ‘CNRA’, included 38 leaf samples of *D. rotundata* collected in 2018 from the yam germplasm collection maintained under open field conditions by the Food Crops Research Station of the Centre National de Recherche Agronomique (CNRA) in Bouaké (Côte d’Ivoire) [10]. Total nucleic acids (TNAs) were purified from each leaf sample using extraction procedure 2, described by Foissac *et al.* [11], and were used as template for cDNA synthesis as described by Umber *et al*. [9]. This was followed by a nested PCR using the five inosine-containing degenerate primers (PDO) designed by Foissac *et al.* [11] (Supplementary Table S2). These primers allow the detection of viruses of the genera *Banmivirus*, *Capillovirus*, *Foveavirus*, and *Trichovirus* (family *Betaflexiviridae*), resulting in the amplification of a 362-bp fragment containing the conserved motifs II, III, IV, and V of the RNA-directed RNA polymerases (RdRps) of positive-stranded RNA viruses [12]. The results of the prevalence survey showed that 24.6% (220/896) of the samples from the ‘Guadeloupe’ sampling tested positive (Table 1). Interestingly, the highest prevalence in this sampling was in plants of the African species *D. cayenensis* and *D. rotundata* (96.2% and 67.8%, respectively). The prevalence was similarly high (78.9%; 30/38) in the ‘CNRA’ sampling, which consisted exclusively of samples from the species *D. rotundata*, but it was significantly lower (14.3%; 58/406) in the ‘BRC-TP’ sampling.

**Table 1:** Prevalence of *Betaflexiviridae* members in yam samples from the three samplings analyzed in this work

Sampling	Guadeloupe							BRC-TP							CNRA						
Yam species	Number of indexed samples	PDO primers		YVY-specific primers		BanMMV-specific primers		Number of indexed samples	PDO primers		YVY-specific primers		BanMMV-specific primers		Number of indexed samples	PDO primers		YVY-specific primers		BanMMV-specific primers	
		N	*%*	N	*%*	N	*%*		N	*%*	N	*%*	N	*%*		N	*%*	N	*%*	N	*%*
*D. alata*	720	135	*18.8*	129	*17.9*	3	*0.4*	141	36	*25.5*	35	*24.8*	1	*0.7*	-	-	*-*	-	*-*	-	*-*
*D. bulbifera*	16	1	*6.3*	1	*6.3*	0	*0.0*	4	1	*25.0*	1	*25.0*	0	*0.0*	-	-	*-*	-	*-*	-	*-*
*D. cayenensis*	26	25	*96.2*	25	*96.2*	0	*0.0*	49	10	*20.4*	7	*14.3*	0	*0.0*	-	-	*-*	-	*-*	-	*-*
*D. esculenta*	31	0	*0.0*	0	*0.0*	0	*0.0*	8	1	*12.5*	1	*12.5*	0	*0.0*	-	-	*-*	-	*-*	-	*-*
*D. rotundata*	87	59	*67.8*	59	*67.8*	0	*0.0*	27	6	*22.2*	5	*18.5*	0	*0.0*	38	30	*78.9*	29	*76.3*	0	*0.0*
*D. trifida*	16	0	*0.0*	0	*0.0*	0	*0.0*	173	4	*2.3*	4	*2.3*	0	*0.0*	-	-	*-*	-	*-*	-	*-*
Other species	-	-	*-*	-	*-*	-	*-*	4	0	*0.0*	0	*0.0*	0	*0.0*	-	-	*-*	-	*-*	-	*-*
Total	896	220	*24.6*	214	*23.9*	3	*0.3*	406	58	*14.3*	53	*13.1*	1	*0.2*	38	30	*78.9*	29	*76.3*	0	*0.0*

N, number of positive samples; %, percentage of positive samples relative to the number of indexed samples

The molecular diversity of yam-infecting members of the family *Betaflexiviridae* was also investigated. For this, 31 amplification products (18, 11, and 2 from the ‘Guadeloupe’, ‘BRC-TP’, and ‘CNRA’ samplings, respectively; Supplementary Table S1) were cloned in pGEM-T Easy Vector (Promega, Charbonnières, France) according to the manufacturer’s protocol. One to six clones per amplification product were randomly chosen and sequenced (Genewiz, Leipzig, Germany), resulting in a total of 80 nucleotide sequences of 310 nt length after primer sequences were removed: 50, 22 and 8 sequences originated from the ’Guadeloupe’, ’BRC-TP’, and ’CNRA’ samplings, respectively (GenBank accession numbers: OP495428–OP495507). This dataset was completed with nucleotide sequences of reference isolates for each of the 15 recognized genera of the family *Betaflexiviridae*, banana virus X (BVX-Som; AY710267; unassigned *Betaflexiviridae*) and YLV-SG1 (KJ789130), sequences available for YVY isolates YVY-Mak and YVY-Dan (MK782910 and MK782911), identified in Nigeria by high-throughput sequencing (HTS) [7], and for dioscorea virus A (DioVA-PE5.1; LC467961), a putative member of family *Betaflexiviridae* reported in yam in Brazil (Hayashi *et al*., unpublished). In addition, sequences from a YVY isolate (IgC10YVYDr) and a BanMMV isolate (IgB10BanMMVDa) identified in a *D. rotundata* plant from Benin and in a *D. alata* plant collected in Guadeloupe, respectively, were also included. These sequences were obtained from contigs assembled *de novo* from yam RNASeq transcriptomic Illumina reads generated by the ARCAD project and referenced under numbers SRX1967863 and SRX1967874, respectively, in the SRA GenBank database [13]. A nucleotide sequence alignment was performed using MAFFT v7.490 [14] and checked visually to ensure that none of the introduced gaps would disrupt conceptually translated protein sequences. A maximum-likelihood phylogenetic tree constructed using IQ-TREE v2.2.0 [15], with the best nucleotide substitution model (TIM3+F+I+G4) chosen by the program based on the Bayesian information criterion, and branch support was estimated with 10,000 ultrafast bootstrap replicates [16] and 1,000 SH-aLRT replicates. The sequence identity matrix was obtained using Clustal Omega v1.2.4 [17].

The phylogenetic analysis confirmed that all 80 sequences determined in this work belonged to viruses of the family *Betaflexiviridae* (Fig. 1). Most of the sequences (77/80) formed a clade distinct from other genera in the family *Betaflexiviridae*. This clade also included YVY-Dan, YVY-Mak, and DioVA-PE5.1 and had the CRB603YVYDc sequence in a basal position. This sequence appeared particularly divergent relative to the other sequences in this clade, sharing with them, on average, only 74.8% nucleotide sequence identity (range, 71.9-77.7%) and 82.3% amino acid sequence identity (79.6-83.5%), while the other sequences in the clade shared, on average, 87.7% nucleotide sequence identity (range 79.0-100.0%) and 96.0% amino sequence acid identity (89.3-100%). Further studies are therefore required to decide on the taxonomic status of the CRB603YVYDc isolate. Analysis of the IgC10YVYDr sequence, which corresponds to a nearly complete viral genome, showed that it shared 96.0% and 94.9% amino acid sequence identity with both YVY-Mak and YVY-Dan isolates in the analyzed complete RdRp and coat protein (CP) region, respectively. Considering that these figures are well above the species demarcation threshold established by the ICTV for the family *Betaflexiviridae* (80% amino acid sequence identity in the RdRp or CP; [18]), IgC10YVYDr has all the hallmarks of a YVY isolate. The other 76 sequences in this clade, which were determined in this study and cover part of the RdRp coding region, shared, on average, 85.6% (range, 82.9-87.7%) and 86.9% (range, 84.2-96.1%) nucleotide sequence identity with those of YVY-Mak and YVY-Dan isolates, respectively (96.9% [94.2-99.0%] and 97.3% [94.2-100%] amino acid sequence identity), and 84.3% (range, 81.3-86.8%) nucleotide sequence identity with that of IgC10YVYDr (92.7% [89.3-95.1%] amino acid identity). Since these identity scores are all in the same range, the 76 sequences generated in this study should most likely also be considered isolates of YVY. The same can be concluded for isolate DioVA-PE5.1, which shared 85.2% and 87.4% nucleotide sequence identity with YVY-Mak and YVY-Dan, respectively (97.1% amino acid sequence identity in the encoded protein for both isolates). The remaining three sequences out of the 80 determined in this study (Kin8BanMMVDa-1, Kin8BanMMVDa-2, and Kin2BanMMVDa), which were obtained from *D. alata* plants of the ‘Guadeloupe’ sampling, shared 79.4-80.0% nucleotide sequence identity and 87.4% amino acid sequence identity with the reference sequence of banana mild mosaic virus (BanMMV; AF314662) and 98.4-99.0% nucleotide sequence identity and 100% amino acid sequence identity with IgB10BanMMVDa. Sequence comparisons of the RdRp and CP proteins from the nearly complete genome sequence of IgB10BanMMVDa and the BanMMV reference isolate showed that IgB10BanMMVDa unambiguously corresponds to a BanMMV isolate (86.3% and 80.2% amino acid sequence identity to AF314662 in the complete RdRp and CP, respectively). These figures strongly support the notion that the three partial sequences determined in this study belong to BanMMV isolates and provide evidence that BanMMV infects *D. alata* in Guadeloupe.

**Fig. 1:**
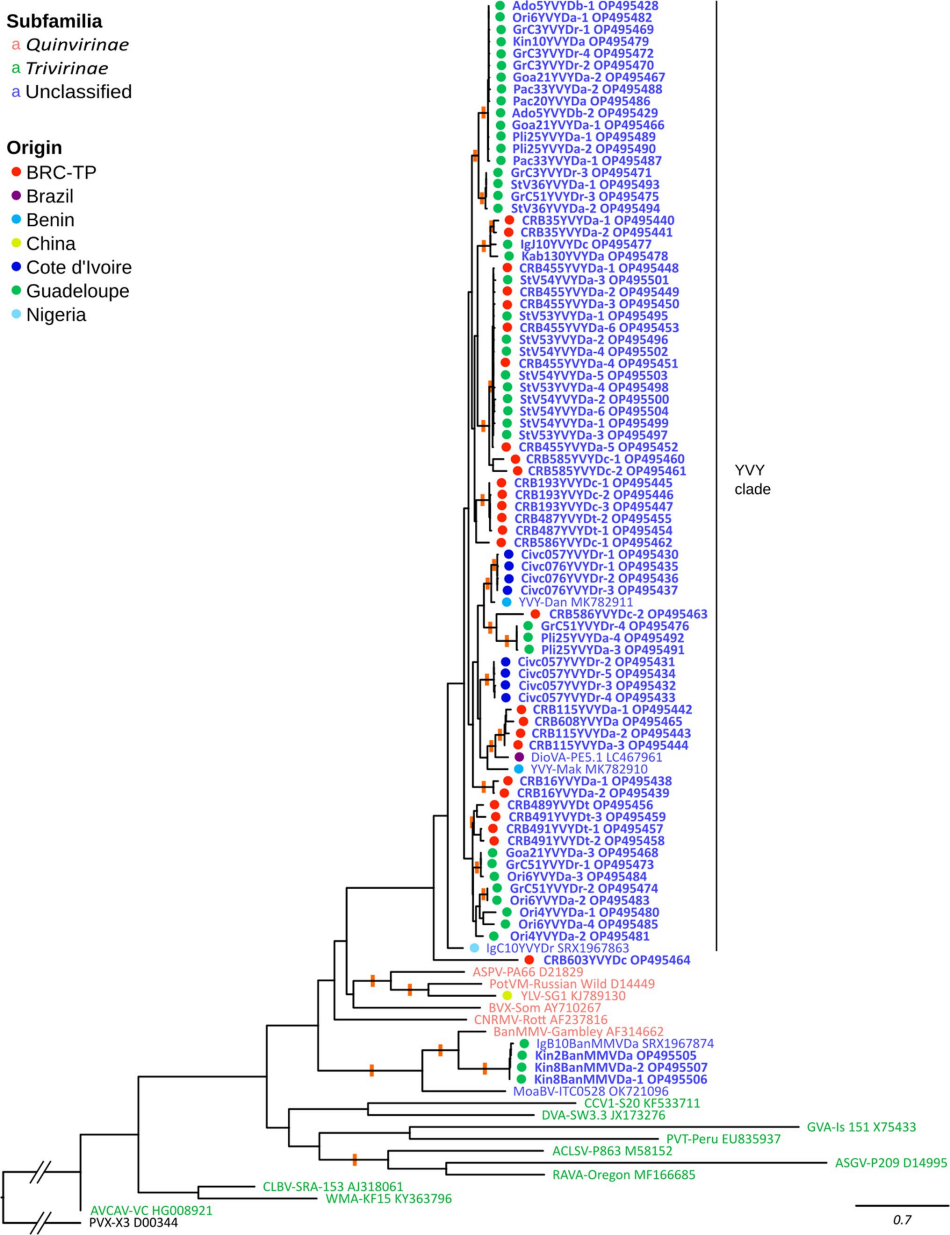
Maximum-likelihood phylogenetic tree showing the relationships between betaflexivirid sequences obtained in this study (in bold) and those of previously characterized isolates. Phylogenetic analysis was performed based on the amplified sequences (310 nt) corresponding to nucleotide positions 4612–4921 of the genome of YVY-Mak and coding for part of the RdRp. Potato virus X (PVX) was used to root the tree. Keys are provided for the coloured sequence names according to their taxonomic position in the family *Betaflexiviridae* (subfamilies recognized by the ICTV), and to the coloured dots referring for the location of the samples from which the sequences originated. Significant branch support (ultrafast bootstrap values above 95% of 10,000 replicates and SH-aLRT above 85% of 1,000 replicates) is indicated by an orange rectangle across the supported branches. Da, *Dioscorea alata*; Db, *D. bulbifera*; Dc, *D. cayenensis*; Dr, *D. rotundata*; Dt, *D. trifida*

The YVY isolates characterized in this work displayed moderately high rates of nucleotide sequence diversity, with an average sequence dissimilarity of 12.9% (0-22.3%), approaching those previously reported for other members of the family *Betaflexiviridae* [11, 19, 20], but with a rather low average amino acid sequence dissimilarity of 3.9% (0-10.7%). Statistical analysis was performed on the 80 YVY sequences used in the phylogenetic analysis described above, including the 76 YVY sequences generated in this work but excluding the CRB603YVYDc sequence, to investigate a possible geographical and/or historical structuring of the molecular diversity of YVY. Four groups of sequences were defined: ‘Africa’ (eight sequences from the ‘CNRA’ sampling, one from Benin, and two from Nigeria), ‘BRC-TP’ (21 sequences), ‘Guadeloupe’ (47 sequences), and ‘S. America’ (one sequence from Brazil). An analysis of molecular variance (AMOVA) was carried out on single nucleotide polymorphisms (SNPs), using R v4.2.1 [21] in order to assess sequence differentiation among these four groups. This analysis revealed a between-group variance of 7.97 (significantly greater than zero; *P* = 0.001), showing that the groups of isolates have diverged from each other. This result was confirmed by a discriminant analysis of principal components (DAPC; Fig. 2), which looks for SNP patterns that maximize between-group distance while minimizing within-group distance. The first discriminant axis appeared to separate the ‘Africa’ group from a metagroup composed of sequences from sources located in the New World (‘BRC-TP’, ‘Guadeloupe’, and ‘S. America’ groups), and the second axis separated the latter three groups, thereby showing that isolates in these groups form genetically differentiated pools. Sequence comparisons revealed an average between-group nucleotide dissimilarity of 14.1-15.2%, while the average within-group dissimilarity was lower (10.2% and 10.5% for ‘Africa’ and ‘Guadeloupe’, respectively), except for the ‘BRC-TP’ group, whose average within-group nucleotide dissimilarity (14.1%) was in the same range as the average between-group dissimilarity.

**Fig. 2:**
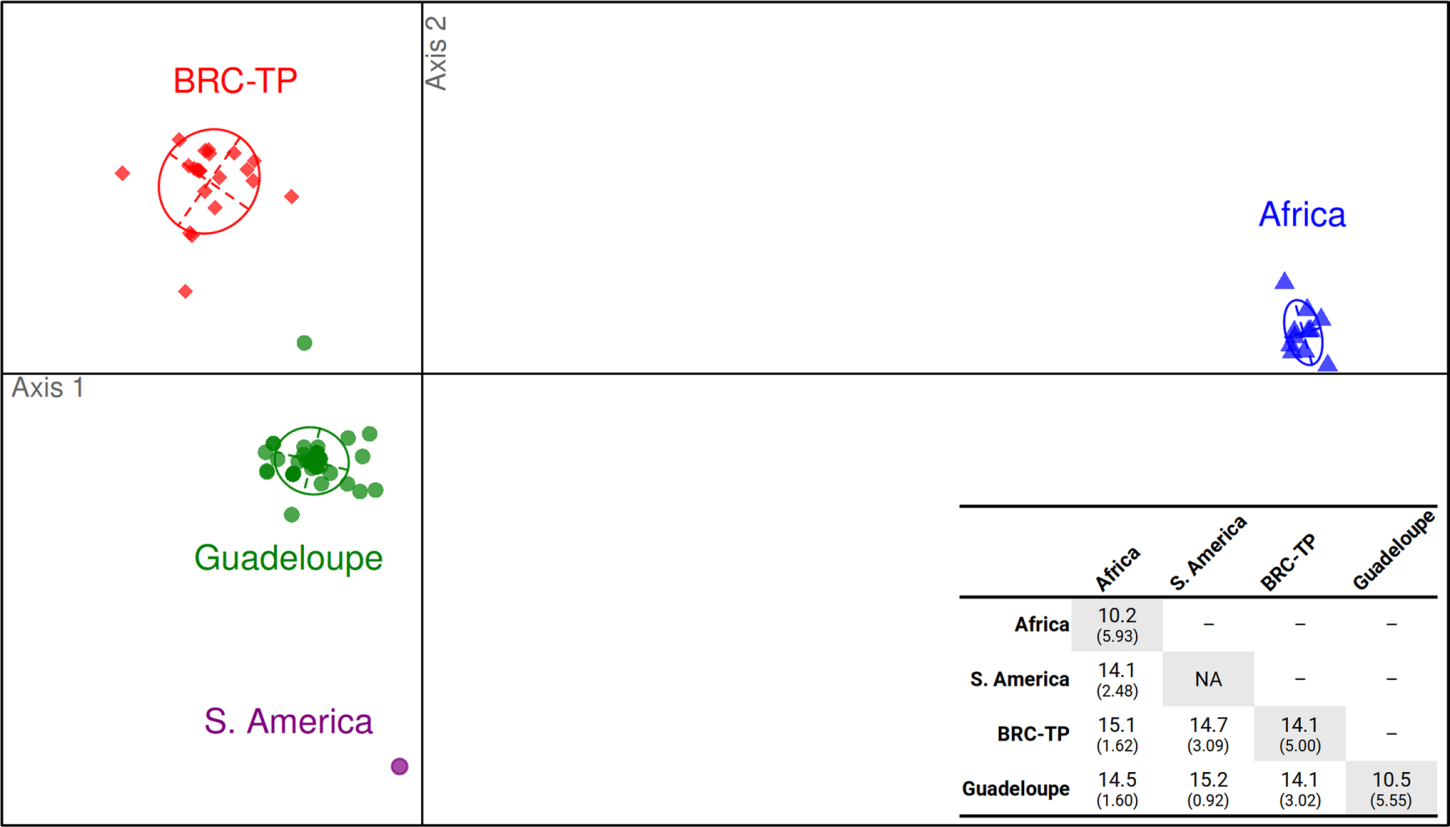
Ordination plot of the discriminant analysis of principal components performed on 76 YVY sequences obtained in this work and four YVY sequences obtained from GenBank. Sequences were assigned to four groups (‘Africa’, ‘BRC-TP’, ‘Guadeloupe’, and ‘S. America’) according to their sampling location and are coloured accordingly on the plot. Ellipses delineate the confidence interval around the group centroids. A between-group percent sequence dissimilarity matrix is shown in the bottom right corner, with the standard deviation in parentheses and within-group dissimilarities shown along the matrix diagonal. NA, not applicable

The prevalence of YVY and BanMMV in the three samplings was investigated. For this, two species-specific primer pairs were designed based on sequence comparisons between all available sequences of YVY and BanMMV yam isolates, in order to capture the molecular diversity of both viruses, and used for virus indexing. Primers YVY-F and YVY-R (Supplementary Table S2) were used in nested RT-PCR experiments, using the PCR products generated from the first PCR step of the PDO-PCR assay as a template. This YVY-specific RT-PCR assay produced amplicons of 275 bp corresponding to nucleotide positions 4640-4914 in the YVY-Mak genome. Conditions for the nested PCR were an initial denaturation step of 2 min at 95° C, followed by 30 cycles of 30 s at 95°C, 30 s at 54°C, and 30 s at 72°C, and a final elongation of 5 min at 72°C. The primers YamBanF and YamBanR (Supplementary Table S2) were used in RT-PCR experiments, producing amplicons of 226 bp corresponding to nucleotide positions 4552-4777 of the BanMMV reference isolate (AF314662). PCR conditions were the same as above except that the annealing temperature was increased to 57°C. Indexing results showed that most of the samples used in this study that were positive with broad-range PDO primers also tested positive when using YVY-specific primers (Table 1; Supplementary Table S1). This proportion was 97.3% for samples of the ‘Guadeloupe’ sampling, 91.4% for those of the ‘BRC-TP’ sampling, and 96.7% for those of the ‘CNRA’ sampling (Table 1), showing the efficiency of this specific detection tool. In contrast, the prevalence of BanMMV was very low, with only 0.3% and 0.2% of the samples from the ‘Guadeloupe’ and ‘BRC-PT’ samplings, respectively, testing positive. All 38 samples from the ‘CNRA’ sampling were negative for BanMMV, strengthening the observation that only *D. alata* seems to be prone to infection by this virus.

Our results extend the known natural host range of YVY to several additional yam species, including *D. alata, D. bulbifera, D. cayenensis*, and *D. trifida* in addition to *D. rotundata*, which had been reported previously [7], and its known geographical distribution to Benin, Côte d'Ivoire and Guadeloupe, where it is widespread. Whether YVY was introduced into Guadeloupe through infected yam imported from Africa during the slave trade [22], as hypothesized for YMV and yam mild mosaic virus (YMMV) [23, 24], remains to be investigated and requires extensive phylogeographical studies with large samplings in Africa. The ‘BRC-TP’ collection includes yam accessions originating from various parts of the world (the Caribbean, South America, Africa, and the South Pacific; Supplementary Table S1) that were introduced before accurate viral diagnostic tools were available, making it impossible to know whether accessions in this collection were infected by YVY prior to their introduction into Guadeloupe or became contaminated while being maintained under field conditions in Guadeloupe prior to their introduction into the ‘BRC-TP’ *in vitro* collection.

The YVY isolates characterized in this work display levels of molecular diversity similar to those reported for other yam-infecting viruses, such as YMV, YMMV, and Dioscorea mosaic associated virus (DMaV) [23, 25, 26]. However, diversity among YVY isolates could be partitioned into four distinct genetic pools by the DAPC analysis (Fig. 2), reflecting a genetic differentiation also seen in the phylogenetic tree in Figure 1 in the form of origin-specific subclades of isolates. The molecular diversity among infected plants was moderate, whereas within-plant molecular diversity was low, contrary to the situation encountered for other yam-infecting viruses such as DMaV [26]. Some YVY sequences with 100% identity were amplified from distinct samples collected from distinct species (Fig. 1), suggesting plant-to-plant transmission of this virus. Whether such transmission occurs mechanically through the use of infected tools for fragmenting yam tubers before planting or involves biological vectors remains to be elucidated. YVY might also be transmitted vertically through vegetative propagation and the use of infected tubers as planting material, as demonstrated for other yam viruses [8]. Since most YVY-infected plants reported in this work were coinfected with other viruses such as YMMV and YaV1 [8, 10], we could not associate YVY with specific symptoms.

The use of primers YVY-F and YVY-R allowed the detection of YVY in 94.2% (290/308) of the samples that tested positive using degenerate PDO primers (Supplementary Table S1), as well as in six samples that tested negative with the PDO primers. On the other hand, 5.8% (18/308) of the samples tested positive when using the PDO primers and negative when using the YVY-specific primers. Four of the latter samples were found to be infected by BanMMV, suggesting that the remaining 14 could have been infected by another virus of the family *Betaflexiviridae*, including potentially uncharacterized YVY variants that escaped detection with the YVY-specific primers described in this work.

Here, we report the first occurrence of BanMMV isolates infecting yam. BanMMV was detected in three *D. alata* samples collected from the same plot, in one *D. alata* hybrid collected in Guadeloupe and used for RNASeq analysis and in one *D. alata* accession from the ‘BRC-TP’ sampling, originating in Vanuatu (CRB767; Supplementary Table S1). The latter was introduced into Guadeloupe in 2003 and maintained under field conditions until it was introduced *in vitro* in 2014. Therefore, it may have been infected by BanMMV either before or after its introduction into Guadeloupe. There is experimental evidence of plant-to-plant transmission of BanMMV in banana in Guadeloupe [19], although the actual mode of transmission (mechanical and/or vector-borne) of this virus has not been elucidated. A possible scenario might involve vector-borne transmission of BanMMV from banana to yam, considering that BanMMV is widespread in banana in Guadeloupe and that both crops are often present together on farm plots, a situation that could favour host jumps from banana to yam.

## Supplementary Information

Below is the link to the electronic supplementary material.

Supplementary file1 (XLSX 92 KB)

Supplementary file2 (DOCX 14 KB)

## Data Availability

The data presented in this study are available in Suplementary Tables S1–S2. The full dataset is openly available at Recherche Data Gouv, V1, 10.57745/GNUXVA, UNF:6:uRiYq5/k7MnOABwsIvhK9A==. The nucleotide sequences reported in this work have been deposited in the GenBank database under accession numbers OP495428—OP495507.
