# Supplementary material for: Molecular diversity of yam virus Y and identification of banana mild mosaic virus isolates infecting yam (Dioscorea spp.)
Source: Arch Virol. 2023 Jun 13;168(7):180. doi: 10.1007/s00705-023-05809-3 (PMC10264530; doi:10.1007/s00705-023-05809-3)
Supplement: Supplementary file 2 — Supplementary file2 (DOCX 14 KB) [file 705_2023_5809_MOESM2_ESM.docx]

**Supplementary Table 2.** Sequences of primers used in this study.

| **Primer name** | **Primer sequence**^a^ | **Reference** |
| --- | --- | --- |
| PDO-F1i | 5’-TiT TYA TKA ARW SiC ARY WiT GiA C-3’ | [11] |
| PDO-F2i | 5’-GCY AAR GCi GGi CAR ACi YTK GCi TG-3’ | [11] |
| PDO-R1i | 5’-TCH CCW GTR AAi CKS ATi Aii GC-3’ | [11] |
| PDO-R3i | 5’GCR CAC ATR TCR TCi CCi GCR AAi iA-3’ | [11] |
| PDO-R4i | 5’-Ari YiC CAT CCR CAR AAM iTi GG-3’ | [11] |
| YVY-F | 5’-GCR CCA ACM ATG MGR TAY ATH GA-3’ | this work |
| YVY-R | 5’-CCR AGT YTY GAR CCC ARR TGW GT-3’ | this work |
| YamBanF | 5’-CTC ACA CAT AGT GTT GTG TAG-3’ | this work |
| YamBanR | 5’-CAT GAT CGC CAC TTC AAA TGC C-3’ | this work |

^a^ The degeneration codes used are as follows: H (A, T, or C); Y (C or T); R (A or G); W (A or T); K (G or T) and i (inosine).
